# Blue and Red Light Modulates SigB-Dependent Gene Transcription, Swimming Motility and Invasiveness in *Listeria monocytogenes*

**DOI:** 10.1371/journal.pone.0016151

**Published:** 2011-01-11

**Authors:** Nicolai Ondrusch, Jürgen Kreft

**Affiliations:** Lehrstuhl für Mikrobiologie - Biozentrum, Universität Würzburg, Würzburg, Germany; Institut de Pharmacologie et de Biologie Structurale, France

## Abstract

**Background:**

In a number of gram-positive bacteria, including *Listeria*, the general stress response is regulated by the alternative sigma factor B (SigB). Common stressors which lead to the activation of SigB and the SigB-dependent regulon are high osmolarity, acid and several more. Recently is has been shown that also blue and red light activates SigB in *Bacillus subtilis*.

**Methodology/Principal Findings:**

By qRT-PCR we analyzed the transcriptional response of the pathogen *L. monocytogenes* to blue and red light in wild type bacteria and in isogenic deletion mutants for the putative blue-light receptor Lmo0799 and the stress sigma factor SigB. It was found that both blue (455 nm) and red (625 nm) light induced the transcription of *sigB* and SigB-dependent genes, this induction was completely abolished in the SigB mutant. The blue-light effect was largely dependent on Lmo0799, proving that this protein is a genuine blue-light receptor. The deletion of *lmo0799* enhanced the red-light effect, the underlying mechanism as well as that of SigB activation by red light remains unknown. Blue light led to an increased transcription of the internalin A/B genes and of bacterial invasiveness for Caco-2 enterocytes. Exposure to blue light also strongly inhibited swimming motility of the bacteria in a Lmo0799- and SigB-dependent manner, red light had no effect there.

**Conclusions/Significance:**

Our data established that visible, in particular blue light is an important environmental signal with an impact on gene expression and physiology of the non-phototrophic bacterium *L. monocytogenes*. In natural environments these effects will result in sometimes random but potentially also cyclic fluctuations of gene activity, depending on the light conditions prevailing in the respective habitat.

## Introduction

*Listeriae* are gram-positive, non-sporulating rod-shaped bacteria. The genus comprises eight species, *L. monocytogenes* and *L. ivanovii* are pathogenic for humans and/or animals, *L. seeligeri* is considered as nonvirulent, *L. innocua*, *L. welshimeri*, *L. grayi*, *L. marthii* and *L. rocourtiae* are harmless saprophytes. Natural habitats of *Listeriae* are decaying plant material in soil and also the intestine of healthy animals and man [1], [2]. From there the bacteria gain access to sewage and water and may also contaminate food processing environments. Uptake of contaminated feed or food leads to the transmission of *Listeria* to mammalian hosts, including man [3], [4].

Listeriosis, a systemic disease caused in humans by *L. monocytogenes*, is rare but has a high mortality in severe manifestations, e.g. sepsis and meningoencephalitis. It mainly occurs in risk groups, such as children, pregnant, elderly and immunocompromised persons [5], [6]. The bacterium has also been implicated in a number of gastroenteritis cases [7]. *L. monocytogenes* is widely studied as model organism for facultative intracellular bacterial pathogens. It turned out that pathogenic *Listeriae* contain a chromosomal cluster of genes (Vcl) which are essential for virulence. The products of these virulence genes are involved in the escape of *Listeria* from the phagosome of the host cell, in actin-based intracellular movement of the bacteria and in their spreading to neighbouring cells [5], [8], [9]. The virulence gene cluster shows some features of a genomic island and therefore has been termed LIPI-1, for *Listeria* pathogenicity island 1 [10], [11]. A steadily growing number of other factors which are involved in the infection process has been identified, among them the internalins which trigger the bacterial uptake into non-phagocytic cells [12]. All virulence genes within LIPI-1 are under the transcriptional control of the Crp-like regulator PrfA [13], [14], [15].

The events during the transition of pathogenic *Listeriae* from the saprophytic lifestyle in the environment to that of an intracellular pathogen have been reviewed recently [16], [17].

*L. monocytogenes* is very robust, growing between pH 5–9, from 1–45°C and at salt concentrations up to 12% [5]. Listerial mechanisms counteracting environmental stress, e.g. high osmolarity, acid and bile, have been studied with respect to the survival in the environment and during the colonization of the intestinal tract [18]–[22]. The alternative stress sigma-factor B (SigB) of the RNA-polymerase holoenzyme was first discovered in the model organism for low G+C *Firmicutes*, *Bacillus subtilis* [23]. It is one of the key components in the general stress response of this group of microorganisms and controls the transcription of a large regulon of stress-related genes [24]. This also holds true for *L. monocytogenes* [19], [25], [26], [27], [28], in addition to SigB the stress gene regulators CtsR [29] and HrcA [30] play an important role. SigB of *L. monocytogenes* is also acting on the transcription of *prfA* and hence of PrfA-dependent genes, thus interconnecting stress response and virulence gene expression [31], [32], [33], [34], [35]. The activation of SigB by stress is a very complex process which has extensively been studied in *B. subtilis*, reviewed in [24]. There it involves, among other factors, the modulator protein RsbR [36]. *B. subtilis* RsbR and its paralogues [37], together with RsbS and RsbT, form a dynamic supramolecular complex, termed stressosome, which is supposed to integrate different environmental stress signals, such as high osmolarity, low pH etc. [38], [39], [40], [41], [42], [43], [44], [45]. Although the constituents of this complex are well characterized, it is not yet really clear which of the RsbR paralogues perceives which kind of environmental signal, with one notable exception. It has been shown recently that YvtA of *B. subtilis* activates SigB upon illumination with blue light [46], [47], [48]. Initially YtvA has been described as a positive regulator of SigB and as a RsbR paralogue with biochemical properties different from other RsbR proteins, i.e. not being phosphorylated by the RsbT kinase in vitro, showing a yellow color upon purification and bearing a N-terminal PAS domain [37]. Subsequent analyses identified YtvA as a member of the newly discovered and growing family of phototropin-related blue-light receptors in prokaryotes [49]. All these proteins carry a N-terminal LOV domain and a variety of C-terminal signalling domains [50]. LOV domains, found in blue-light receptors, oxygen-sensors and voltage-gated potassium channel proteins, are a subfamily of the PAS superfamily. Photoactive LOV domains non-covalently bind FMN (flavin mononucleotide), upon illumination with blue light a covalent photoadduct of a single molecule of FMN to a conserved cysteine in the LOV domain is formed and a photocycle is initiated which ultimately leads to a signal transduction process [51]. In YtvA the LOV domain is linked to a C-terminal STAS domain, the latter is characteristic for sulphate transporters and anti-sigma-factor antagonists [52] and is present in all RsbR paralogues and in RsbS of *B. subtilis* [37], [41]. The photochemistry and structure of YtvA has been studied in great detail [53], [54], [55], [56], [57] as well as its possible mechanism of action in SigB activation [58]. In these studies it has firmly been established that YtvA is a true flavin-based photoreceptor. Recently it has been described that the SigB-mediated general stress response of *B. subtilis* is also activated by red light, however, a receptor has not unambiguously been identified [59]. It has been noted early that proteins with the particular domain architecture of *B. subtilis* YtvA, i.e. N-terminal LOV linked to C-terminal STAS, are only found in other *Bacilli* and in *Listeria* [50], [51], however, nothing was known so far about the function of Lmo0799, the presumptive homologue of YtvA in the pathogen *L. monocytogenes*. Since a long time light-induced physiological responses and signalling processes are well known for photosynthetic microorganisms, but reports on such phenomena in non-phototrophic bacteria were rather rare, this has changed in the last decade [49], [50], [60], [61], [62], [63], [64], in particular since an exponentially growing number of fully sequenced bacterial genomes became available [65], [66], [67]. However, still very little is known about light effects on bacterial pathogenesis [68]. It has been shown that light influences the virulence of the plant pathogen *Agrobacterium tumefaciens* [69]. In the plant pathogen *Pseudomonas syringae* a blue light inducible two-component system has been characterized [70], it is not known if this system has a role in pathogenicity. With respect to bacteria pathogenic for mammalians it has been reported that infection of macrophages by *Brucella abortus* was stimulated by blue light, this effect was dependent on a photoreceptor which combines a LOV domain with a histidine-kinase signalling domain [71]. Here we demonstrate for the first time that the Lmo0799 protein of *L. monocytogenes* EGD-e is indeed a functional homologue of the photoreceptor YtvA from *B. subtilis*. We show that it is involved in the blue-light driven transcriptional activation of SigB-regulated genes in *L. monocytogenes* and that blue-light activation of Lmo0799 impaired the swimming motility of the bacteria. Among the light-induced genes were also those for the internalins A and B, resulting in an increase of bacterial invasiveness for enterocyte-like Caco-2 cells. Furthermore, we could demonstrate that blue light had a Lmo0799-independent effect on the transcription of stress- and virulence-related genes of *L. monocytogenes* and that transcription of these genes was also influenced by red light, the underlying mechanisms remain unknown. Light induction of SigB-dependent genes was completely abolished in a sigma B deletion mutant.

## Results

Lmo0799 *of Listeria monocytogenes* EGD-e is highly similar to YtvA from *Bacillus subtilis*, homologues are present in all sequenced *L. monocytogenes* isolates and in other *Listeria* species.

**Figure 1:**
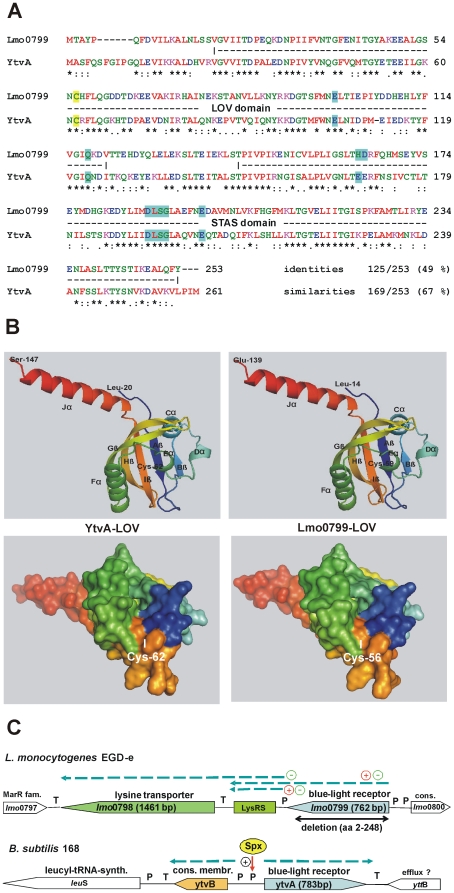
Structure and genetic organization of Lmo0799 and YtvA. Detailed explanations in the text. (A) Amino acid sequence alignment of Lmo0799 and YtvA, using ClustalW2 [106] (http://www.ebi.ac.uk/Tools/clustalw2/index.html). Asterisks below the sequence indicate identical, double points very similar amino acids. The boundaries of the LOV and STAS domains are according to [56]. Amino acids which have been shown to be particularly important for the function of YtvA are highlighted in yellow (C62/C56) or blue. (B) 3-D models of YtvA-LOV and Lmo0799-LOV, generated with the SWISS-MODEL server (http://swissmodel.expasy.org/workspace/). Upper panel: Ribbon models, the designations of beta-sheets and alpha-helices were from [56]. Lower panel: Surface models. (C) Genomic context and transcriptional organization of *lmo0799* and *ytvA*. The data for *lmo0799* were from [35], for *ytvA* from SubtiList (http://genolist.pasteur.fr/SubtiList) and from [47]. P: promoter, T: transcriptional terminator. The GenBank accession nos. for the respective genome sequences are AL591824 (*L.m*.) and AL009126 (*B.s*.).

During a recent investigation into the thiol:disulfide redox metabolism of *Listeria monocytogenes* EGD-e we observed that, together with the genes for thioredoxin A (*trxA*) and thioredoxin reductase (*trxB*), the transcription of *lmo0799* was highly up regulated when the bacteria were selectively depleted for the biological thiol glutathione (GSH) by a deletion in the glutathione synthetase gene *gshF*. Treatment of *L. monocytogenes* with diamide, an oxidant for biological thiols (not only GSH but also thiol-containing proteins), did not significantly induce *lmo0799* (unpublished results). The gene product of *lmo0799* has originally [72] been annotated as a hypothetical protein of 253 amino acids (http://genolist.pasteur.fr/ListiList/), however, a closer look revealed a significant relatedness to the blue-light photoreceptor YtvA of *B. subtilis,* this had already been noted earlier by others [50], [51]. Figure 1A shows that the two amino acid sequences are highly similar over their total length, this includes the cysteine (C62 in YtvA, C56 in Lmo0799) which is required for formation of the FMN photoadduct. As already mentioned in the introduction, YtvA of *B. subtilis* is a RsbR paralogue. However, it lacks the conserved phosphorylatable threonines found at positions 171 and 205 in RsbRA and at corresponding positions in RsbRB-D [37], [73]. In YtvA negatively charged glutamates (E168 and E202) are present at positions corresponding to T171/T205 of RsbRA. It has been suggested that these negatively charged amino acids mimick the phosphorylated state of threonine [47]. In Lmo0799 a histidine (H163) is found at the position of YtvA-E168, followed by a negatively charged aspartate (D164). The second glutamate, E202 in YtvA, is conserved in Lmo0799 (E197). Other amino acids which have been identified as being important for the function of YtvA, e.g. E105, Q123 and the DLSG motif [57], [58], [74] are conserved in Lmo0799. The 3-D structure of the LOV domain of YtvA from *B. amyloliquefaciens* has recently been solved [75], using this structure as a template we modeled the probable structure of Lmo0799-LOV at the SWISS-MODEL workspace. Figure 1B shows that the modeled 3-D structures of YtvA-LOV and Lmo0799-LOV are virtually identical, the coil connecting Hβ and Iβ of Lmo0799-LOV could not be modeled unambiguously. Modeling of the surface of both proteins showed that the crucial cysteines at the N-terminus of Eα are located in a pocket but are accessible in both proteins. Figure 1C depicts the transcriptional organization of the *lmo0799*/*ytvA* loci, as deduced from the literature and from genome data. For *lmo0799* it has been shown by Toledo-Arana et al. [35] that a riboswitch (LysRS), regulating the transcription of the lysine transporter Lmo0798, is located downstream of *lmo0799* in *L. monocytogenes* EGD-e. In the above mentioned study it was shown that both in the absence or presence of lysine in the medium, *lmo0799* was transcribed from a promoter immediately upstream of the gene, this transcript extended into the riboswitch sequence. In addition, a short transcript comprising the riboswitch only was also found. The lysine transporter gene *lmo0789* was only transcribed in the absence of lysine. The graphical representation of the *B. subtilis* 168 genome [76] which is available on a website at the Pasteur Institute Paris (http://genolist.pasteur.fr/SubtiList) clearly indicated that *ytvA* is transcribed monocistronically, its promoter has been mapped by Gaidenko et al. [47].

When we performed a BLAST search, using Lmo0799 as the query sequence, in 1299 bacterial genomes in the NCBI database, our results confirmed previous report, based on a smaller number of genomes [50], [51], that proteins with the particular domain architecture of YtvA/Lmo0799 can be found only in other *Bacilli*, including the extremely halotolerant and alkaliphilic deep-sea isolate *Oceanobacillus iheyensis*, and in *Listeria*. In this search and also using the Genolist website at the Pasteur Institute Paris we found identical or very similar proteins in all sequenced *L. monocytogenes* isolates and *Listeria* species (alignments not shown). The similarity values (identical/positive, in percent) were 99/99 for *L. monocytogenes* serovar 4b strains, 93/99 for *L. innocua*, 91/97 for *L. welshimeri*, 87/96 for *L. ivanovii* (searched at http://genolist.pasteur.fr/LivaList), 85/95 for *L. seeligeri* and 64/80 for *L. grayi*. These values are in agreement with the established phylogenetic tree of *Listeria* which classifies *L. monocytogenes* and *L. innocua* as belonging to one group, *L. ivanovii* and *L. welshimeri* to another and *L. grayi* as the most distantly related one to all other *Listeria* species [11], [77].

### A deletion mutant for *lmo0799* growths like wild type

By allel replacement a mutant (Δ*0799*) of *L. monocytogenes* EGD-e was constructed in which the coding sequence for the amino acids 2–248 of the 253 amino acids long Lmo0799 protein was deleted (Figure 1C). Such an in frame deletion should not influence the expression of the riboswitch LysRS and of the lysine transporter Lmo0798. When wildtype or the mutant Δ*0799* were grown in BHI at 37°C no difference in their multiplication was observed (data not shown).

### Blue and red light, in combination with salt stress, induce the transcription of the SigB-dependent *ctc* gene and of *sigB*

**Figure 2:**
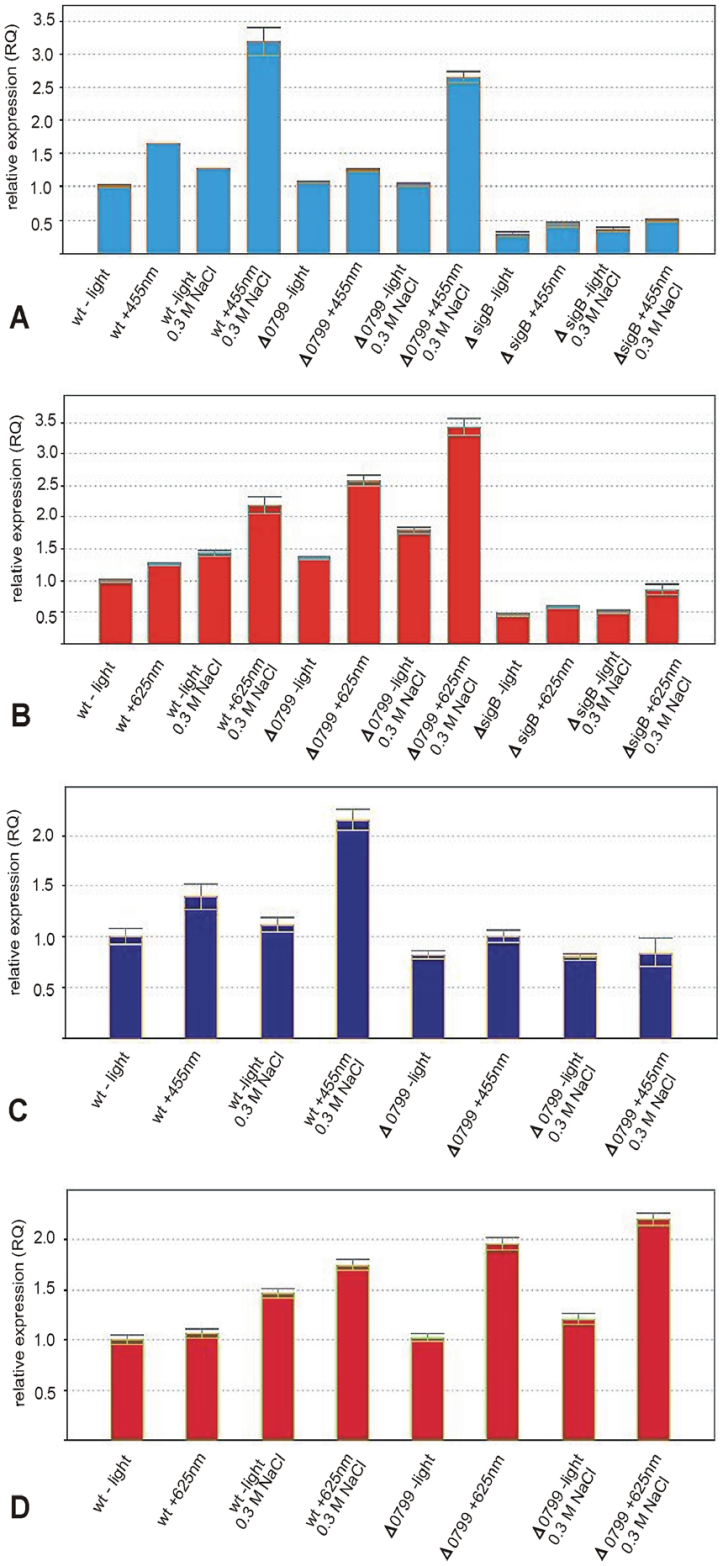
Effect of blue and red light on *ctc* expression. Transcription analysis by qRT-PCR for wild type, Δ*0799* and *ΔsigB* mutants. The strains were grown at 37°C in BHI. Cells were harvested in mid-log phase (OD_600_ ∼0.9) and exposed for 10 min to blue (455 nm) or red (625 nm) light as described in material and methods. The results from the qRT-PCR analysis, obtained with a StepOnePlus Real-Time PCR system (Applied Biosystems Inc.) were normalized using *rpoB* as an internal standard [103], [104] and expressed as fold change with the values for wild type without light set as 1.0. Calculations were performed with the StepOne Software v2.1 (Applied Biosystems Inc.). Means and standard deviations from three independent biological samples and four technical replicates per sample.

In the studies on YtvA of *B. subtilis* the *ctc* gene was used as a reporter gene [46], [47], [48]. The promoter of this general stress gene is the best studied SigB-dependent one in *B. subtilis*, a homologue (*lmo0211*) is also present in the genome of *L. monocytogenes* EGD-e. It has been shown that Ctc of *L. monocytogenes* is involved in osmotolerance [78], in almost all studies on the SigB regulon of *L. monocytogenes* the *ctc* gene appeared as being positively regulated by SigB [32], [79], [80], [81], [82]. In the initial study on the blue-light mediated effect of YtvA it was claimed that red light had no effect on the transcription of *ctc* in *B. subtilis* [46]. However, our preliminary experiments concerning effects of blue light on *L. monocytogenes* yielded highly variable and sometimes conflicting results when we used a red-light dark chamber lamp for the handling of dark controls (not shown). Therefore we used low-intensity, diffuse infrared (λ = 850 nm) illumination for our dark controls in all experiments reported here and night vision goggles for observation during the manipulations. Our assumption that red light might have an effect on transcription was later confirmed for *B. subtilis* too [59]. For YtvA is has been demonstrated that the blue-light effect was enhanced upon exposure of *B. subtilis* to salt stress (0.3 M NaCl) [46], [47], [48], therefore we performed all our transcription analyses after exposure of *Listeria* to all possible combinations of light and salt stress. Figure 2A shows that blue light alone moderately increased *ctc* transcription in the wild type (1.5-fold), the effect of salt stress alone was not significant here as well as in the Δ*0799* receptor mutant, blue light alone had no effect on the mutant. The combination of blue light and 0.3 M NaCl resulted in a 3-fold increase for the wild type and 2.5-fold for the mutant. The response profile for red light was different (Figure 2B), light or salt stress alone resulting in no significant increase for the wild type, red light plus 0.3 M NaCl yielding a 2.2-fold increase here. Surprisingly, red light alone resulted in a 2.5-fold increase in the Δ*0799* mutant, red light plus salt gave a 3.5-fold increase, i.e. significantly higher than in the wild type. Deletion of *sigB* (Δ*sigB*) decreased the transcription of *ctc*, under all conditions, to about half of the level observed for the wild type without light (Figures 2A and 2B). The transcription of *sigB* itself, which is positively autoregulated [26], [82] showed the same pattern as *ctc,* except for the Δ*0779* mutant with blue salt plus salt, where no induction was seen (Figures 2C and 2D).

### Other SigB-dependent genes respond to light in a manner similar to *ctc*, PrfA-regulated virulence genes and the thiol redox-related gene *trxA* behave differently

**Figure 3:**
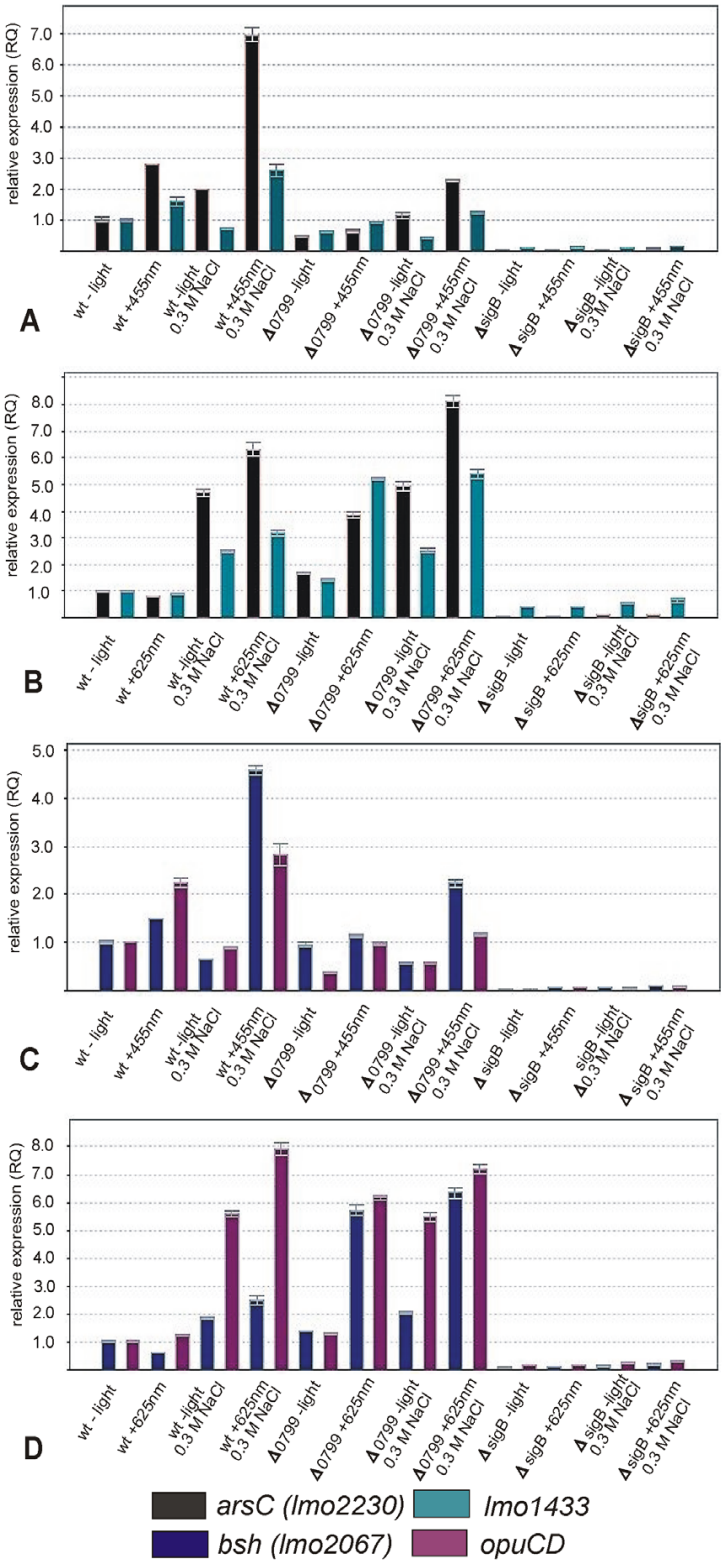
Effect of blue and red light on expression of *arsC*, *lmo1433*, *bsh* and *opuCD*. Transcription analysis by qRT-PCR for wild type, Δ*0799* and *ΔsigB* mutants. The strains were grown at 37°C in BHI. Cells were harvested in mid-log phase (OD_600_ ∼0.9) and exposed for 10 min to blue (455 nm) or red (625 nm) light as described in material and methods. The results from the qRT-PCR analysis, obtained with a StepOnePlus Real-Time PCR system (Applied Biosystems Inc.) were normalized using *rpoB* as an internal standard [103], [104] and expressed as fold change with the values for wild type without light set as 1.0. Calculations were performed with the StepOne Software v2.1 (Applied Biosystems Inc.). Means and standard deviations from three independent biological samples and four technical replicates per sample.

After having established the proper experimental conditions for dark controls, we next investigated the behavior of four other genes the transcription of which is commonly regarded as being SigB-dependent in *L. monocytogenes* [32], [79], [80], [81]. These were *lmo2230* (*arsC*, enconding a protein with similarity to arsenate reductase), *lmo1433* (putative glutathione reductase), *lmo2067* (*bsh*, bile salt hydrolase) and *lmo1425* (*opuCD*). The latter gene encodes one of the two membrane-spanning proteins of a carnitine-ABC-transporter involved in osmo- and bile-tolerance of *L. monocytogenes* [22]. *Lmo1425* was chosen instead of *lmo1428* (*opuCA*), the first gene in the *opuC* operon, because in this way we could better monitor the transcription of the entire operon. Figure 3 shows that the light- and salt-dependent transcription profiles of all these genes were similar to that observed for *ctc*. Induction by blue light was always strongest in combination with salt stress and was significantly reduced or abolished when Lmo0977 was lacking (Figures 3A and 3C). In wild type bacteria an induction by red light was again only observed in combination with salt stress whereas in the Δ*0799* mutant all four genes were induced by red light also in the absence of salt stress (Figures 3B and 3D). In contrast to *ctc* the deletion of *sigB* decreased the transcription to almost zero under all conditions.

**Figure 4:**
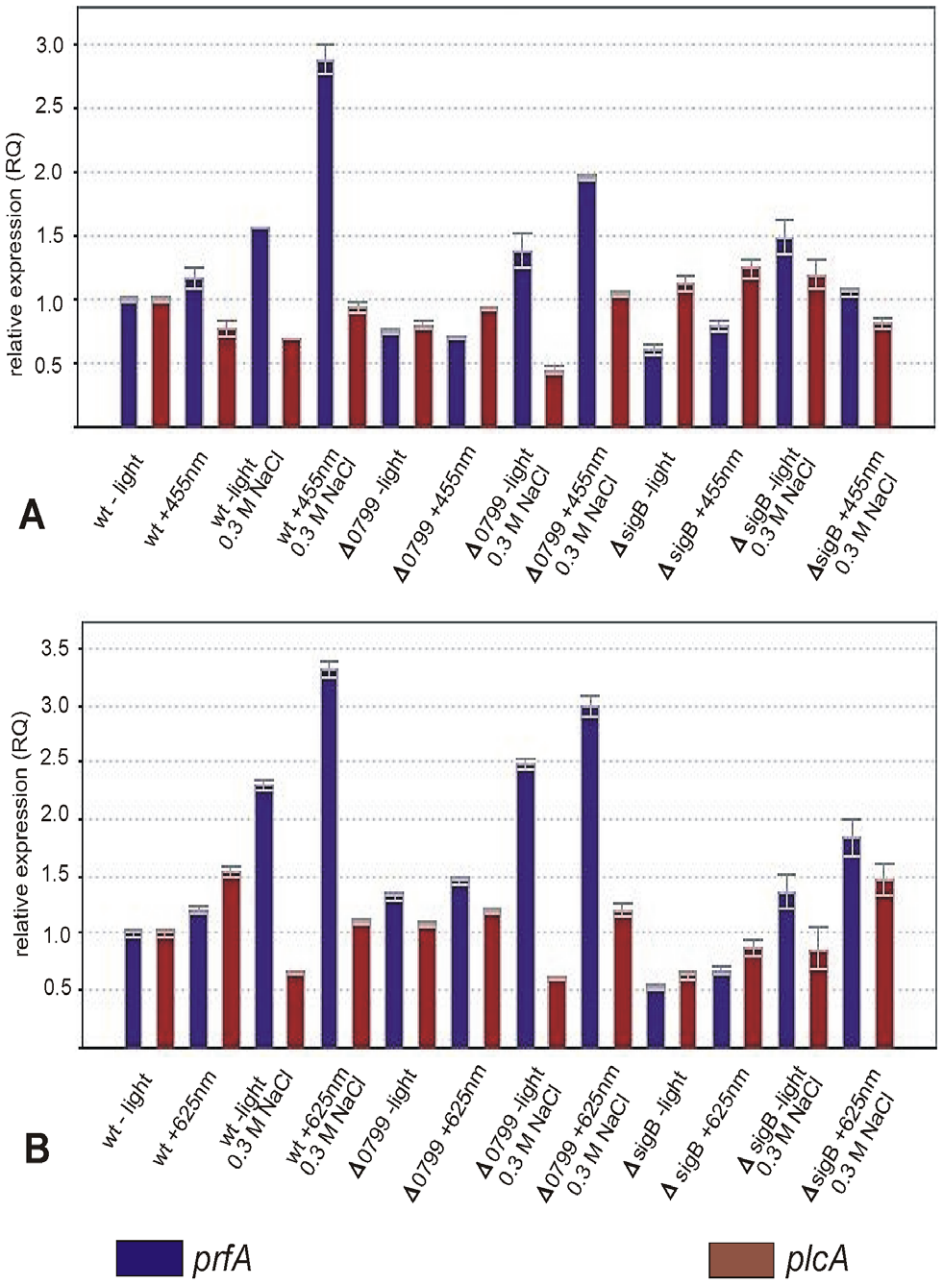
Transcription profiles of virulence genes. Transcription analysis by qRT-PCR for wild type, Δ*0799* and *ΔsigB* mutants. The strains were grown at 37°C in BHI. Cells were harvested in mid-log phase (OD_600_ ∼0.9) and exposed for 10 min to blue (455 nm) or red (625 nm) light as described in material and methods. The results from the qRT-PCR analysis, obtained with a StepOnePlus Real-Time PCR system (Applied Biosystems Inc.) were normalized using *rpoB* as an internal standard [103], [104] and expressed as fold change with the values for wild type without light set as 1.0. Calculations were performed with the StepOne Software v2.1 (Applied Biosystems Inc.). Means and standard deviations from three independent biological samples and four technical replicates per sample.

As figures 4A and 4B show the transcription profiles of *prfA* (encoding the master regulator of virulence in *L. monocytogenes*) and of the PrfA-regulated virulence gene *plcA* exhibited clear differences to canonical SigB-regulated genes. *PrfA* transcription was slightly induced by 0.3 M NaCl in wild type bacteria and in the *Δ0799* mutant without light and was further increased under combined stress conditions (blue or red light plus salt). *PrfA* showed neither a significant induction nor repression in the Δ*sigB* mutant, the transcription of *plcA* was not significantly altered in all strains and under all conditions tested.

The transcription of the thiol redox-related gene *trxA* (*lmo1233,* thioredoxin A) was moderately induced by salt stress in the wild type and the Δ*0799* mutant, no further increase was caused by blue or red light. For blue, but not for red light these effects were less in the Δ*sigB* strain (Figure S1).

### Blue light induces the transcription of the internalin A and B genes and increases the invasiveness of *L. monocytogenes* EGD-e for Caco-2 enterocytes

**Figure 5:**
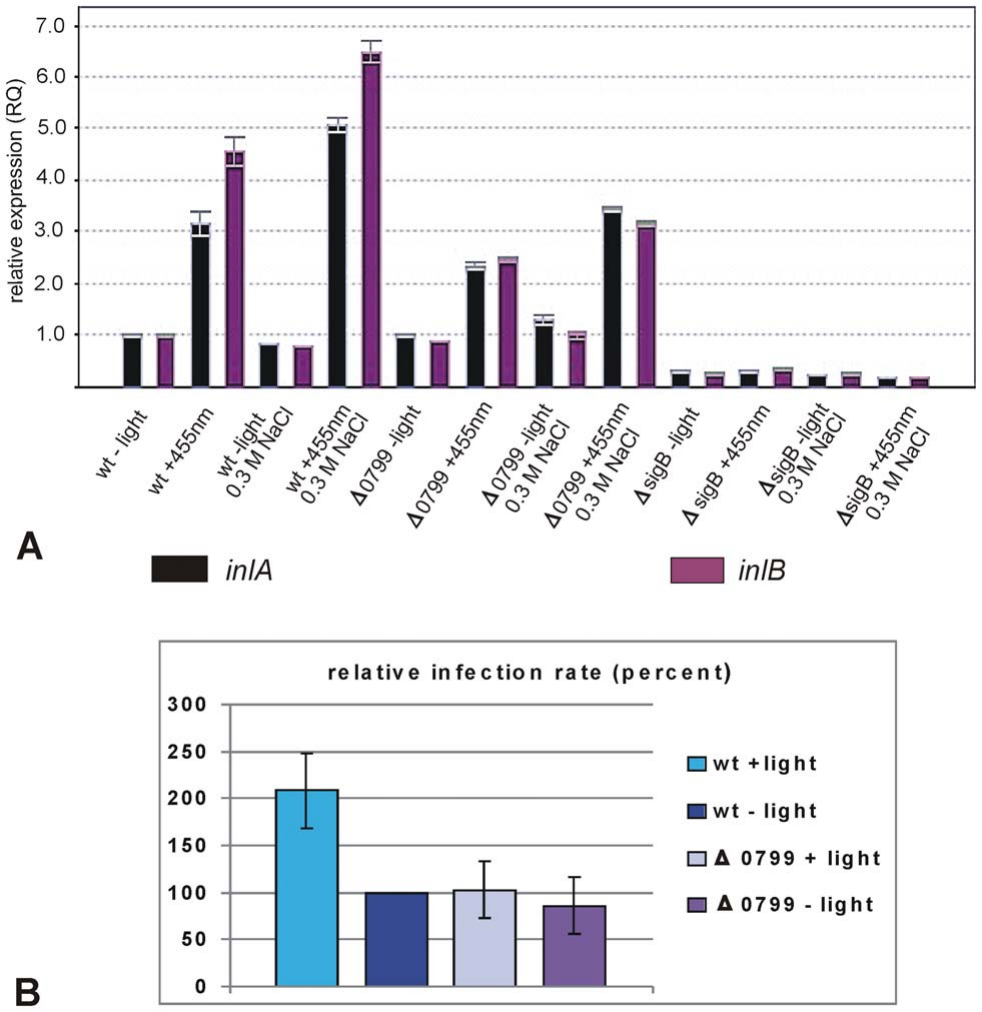
Effect of blue light on *inlA* and *inlB* transcription and on infection rate. (A) Transcription analysis by qRT-PCR for wild type, Δ*0799* and *ΔsigB* mutants. The strains were grown at 37°C in BHI. Cells were harvested in mid-log phase (OD_600_ ∼0.9) and exposed for 10 min to blue light (455 nm) as described in material and methods. The results from the qRT-PCR analysis, obtained with a StepOnePlus Real-Time PCR system (Applied Biosystems Inc.) were normalized using *rpoB* as an internal standard [103], [104] and expressed as fold change with the values for wild type without light set as 1.0. Calculations were performed with the StepOne Software v2.1 (Applied Biosystems Inc.). Means and standard deviations from three independent biological samples and four technical replicates per sample. (B) Caco-2 enterocyte-like cells were infected (m.o.i = 20) with wild type and its isogenic *Δlmo0799* mutant. Prior to infection the bacteria were either exposed for 10 min to blue light or kept in the dark, in both cases at 37°C and with 0.3 M NaCl in the medium. For details see materials and methods. One hour after the addition of gentamicin the cells were lysed, CFU/ml were determined and the relative infection rates were calculated. Means and standard deviations from four independent experiments. The values for wild type without light were set as 100 percent, therefore no standard deviation is shown there.

Since long it has been established by others that the invasion of normally non-phagocytic eukaryotic host cells by *L. monocytogenes* depends on the leucine-rich repeat proteins of the internalin superfamily. Internalin A (InlA, Lmo0433) is required for the invasion of enterocytes, the *inlA* gene is co-transcribed with the downstream gene for internalin B (InlB, Lmo0434) which recognizes other host cell receptors and cell types, reviewed in [8], [9], [12] Figure 5A shows that the transcription of *inlA* and *inlB* was significantly induced (3 and 4.5-fold, respectively) by blue light in the case of the *L. monocytogenes* EGD-e wild type. When blue light was combined with salt stress, *inlA* transcription was further increased to 5-fold, that of *inlB* to 6.5-fold. In the Δ*0799* receptor mutant the induction of both genes by light or light plus salt stress was only 2.5 and 3.5-fold, respectively. Deletion of *sigB* resulted in an almost undetectable transcription under all conditions. *L. monocytogenes* EGD-e wild type and its *Δ0799* receptor mutant were grown at 37°C and in the dark until mid-log phase (OD_600_ = 0.9) with BHI as medium, then one aliquot was exposed to blue light plus 0.3 M NaCl for 10 min at 37°C, the other aliquot was also adjusted to 0.3 M NaCl but kept in the dark under otherwise identical conditions. Semi-confluent Caco-2 enterocyte-like human cells were infected (m.o.i. of 20) with the differently pretreated bacteria for 2 hours (1 hour attachment time in the dark without antibiotic, washing step, followed by one hour incubation with gentamicin). The absolute infection rates (c.f.u./ml lysate) obtained in independent experiments were rather variable, therefore the means and standard deviations of the relative infection rates were used for comparison, setting the values for the wild type without light as hundred percent. As Figure 5B shows an exposure of the bacteria to blue light and 0.3 M NaCl for 10 min resulted in a two-fold increased infectivity of the wild type, when compared to the dark control, whereas infectivity of the Δ*0799* receptor mutant was not significantly changed by blue light. It has repeatedly shown by others that the invasiveness of a Δ*sigB* mutant is drastically reduced [19], [31], [94], therefore this mutant was not tested here. Since the effect of red light on *inlA* and *inlB* transcription basically was identical to that of blue light (results not shown), no infection experiments were performed with red light-exposed bacteria.

### Blue, but not red light inhibits swimming motility of *L. monocytogenes* EGD-e in a Lmo0799- and SigB-dependent manner

**Figure 6:**
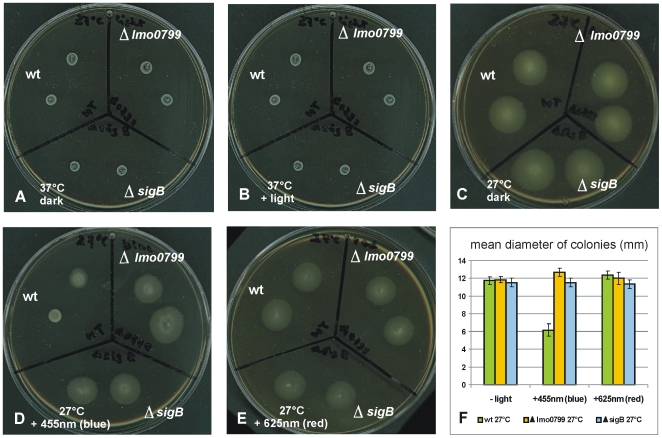
Effect of incubation temperature and light on swimming motility. Semi-solid BHI agar (0.3%) plates were inoculated with 2 µl of mid-log cultures (OD_600_ ∼0.7) of wild type, *Δlmo0799* and *ΔsigB* mutants. Incubation was for 18 h at temperatures and light conditions as indicated in the figure. (B) shows the effect of blue light, the result for red light was identical, (F) shows the means and standard deviations of the colony diameters from three independent experiments.

Assays for swimming motility on semi-solid (0.3 percent agar) BHI plates showed that *L. monocytogenes* EGD-e wild type and its Δ*0799* or Δ*sigB* mutants were non-motile at 37°C, with a colony size of just 6 mm after 16 h incubation time (Figures 6A and 6B). When incubated for 18 h at 27°C in the dark, all strains were clearly motile, reaching colony diameters of about 12 mm (Figures 6C and 6F). Upon constant illumination for 18 h with blue light at an incubation temperature of 27°C, wild type bacteria were almost as non-motile as at 37°C, with a colony diameter of 6 mm and a very faint halo surrounding the colonies (Figures 6D and 6F). The inhibition of swimming motility by blue light was completely relieved in the Δ*0799* blue-light receptor and in the Δ*sigB* mutant (Figures 6D and 6F), no inhibition was observed after exposure to red light of the same intensity (Figures 6E and 6F).

## Discussion

As has already been noticed by others some time ago, *Listeria monocytogenes, L. innocua* [50], [51] and other *Listeria* species [57] contain a gene, *lmo0799* in *L. monocytogenes* EGD-e, which encodes a protein with high similarity to the well characterized blue-light photoreceptor YtvA of *B. subtilis*. Figure 1A depicts the sequence similarities between the two proteins, see also results. Our modeling of the light-receiving domain Lmo0799-LOV, based on the experimentally determined structure of YtvA-LOV [75], revealed that Lmo0799-LOV most probably has the same three-dimensional structure as LOV in the *Bacillus* protein (Figure 1B).

In order to test experimentally if Lmo0799 is a functional blue-light receptor we constructed a strain (Δ*0799*) carrying an in-frame deletion, this mutant was not impaired in growth in BHI. The appropriate test conditions for light exposure experiments were based on the following considerations. For the photocycle of *B. subtilis* YtvA is has been shown that the time required for light activation (conversion of the dark state YtvA_447_ into the red-shifted intermediate YtvA_660_) and for photoadduct formation (YtvA_390_) is very short, 20 nsec and 2 µsec, respectively, whereas the dark recovery is unusually slow, with a Tau_rec_ of 2600 sec (ca. 43 min) [55]. We assumed that the kinetics of the postulated photocycle of Lmo0799 would be in the same time range, allowing to monitor effects after the inevitable interval (about 25 min) between exposure to light and experimental measurements. Since our preliminary experiments had indicated that even low-intensity red light had an effect on gene transcription our dark controls were handled without any visible light. Furthermore, all experiments were done in parallel with blue (λ = 455 nm) and red (λ = 625 nm) light.

Our qRT-PCR results showed that a 10 min exposure of *L. monocytogenes* wild type to blue light and 0.3 M NaCl lead to a three-fold induction of the transcription of *ctc*, the traditional *sigB* reporter gene in *B. subtilis*. This effect was only slightly reduced in the *Δ0799* photoreceptor mutant and also the *ΔsigB* mutant still showed a substantial transcription of *ctc* (Figure 2A). This shows that in *L. monocytogenes ctc* is not strongly dependent on SigB, which is in line with a previous report by others [79]. The transcription of the autoregulated [26], [82] *sigB* gene itself was only induced by the combination of blue light and salt stress, this induction was dependent on the presence of Lmo0799 (Figure 2C). The transcription analysis of the four genes *arsC*, *lmo1433*, *bsh* and *opuCD,* which are representative for the SigB regulon of *L. monocytogenes* [32], [79], [80], [81], showed another patten (Figure 3). For blue light alone the induction was more than two-fold only for *arsC* and *opuCD*, 0.3 M NaCl alone had almost no effect on all four genes. The blue light plus salt effect was abolished in the Δ*0799* deletion mutant in the case of *lmo1433* and *opuCD* and significantly lowered for *arsC* and *bsh*. This is in line with previous reports which have demonstrated that *arsC* and *bsh* showed a very strong induction upon activation of the SigB system, whereas *lmo1433* and *opuCD* were only moderately induced [32], [34], [79], [80], [81]. Deletion of *sigB* completely abolished the transcription of all four genes under all conditions (Figure 3). Together these results showed that i) blue light or salt alone had a stimulatory effect on those genes only which require a low level of active SigB, e.g. *arsC* and *opuCD*, ii) full induction required both blue light and salt stress and was dependent on Lmo0799, iii) the blue light and osmotic effects were strictly dependent on SigB. Thus we have firmly established that Lmo0799 is a genuine blue-light receptor and a functional homologue of YtvA.

Furthermore, our results demonstrated that red light too activated the transcription of SigB-regulated genes in *L. monocytogenes*. Such a red-light effect on SigB activity has recently also been reported for *B. subtilis* and the results presented there showed that the *B. subtilis* proteins RsbP/Q were required to transduce the red-light signal to the SigB cascade, it has been proposed that RsbP was the light-sensing protein [59]. In *B. subtilis* RsbP/Q are part of the energy stress pathway of SigB activation which responds to changes in the intracellular ATP level [24]. In *L. monocytogenes* the perception and transduction of the red-light signal must be different from the mechanism proposed for *B. subtilis* because no homologues of RsbP/Q can be found in *Listeria* [27], [82]. The prototype of red-light photoceptors are the phytochromes [83], also found in many prokaryotes [60], [61], [84] The bacteriophytochromes all share a domain required for the binding of a bilin chromophore [85]. However, proteins with a phytochrome-like structure could not be detected in *Listeria* (and *Bacillus*) genomes during expert bioinformatic analyses published by others [67], [85]. It should be noted, however, that acccording to its genome annotation [72] (http://genolist.pasteur.fr/ListiList) *L. monocytogenes* EGD-e contains all genes required for heme biosynthesis and also for heme oxygenase (*ctaA*, *lmo2058*), required for the conversion of heme into biliverdin, however, a bilin-binding photoreceptor protein could not be identified. Although bilins in their photo-excited singlet- or triplet state can react with molecular oxygen giving rise to superoxides [86], [87], [88], we have no evidence for the generation of substantial oxidative stress in *L. monocytogenes* by red (or blue) light. As figure S1 shows neither blue nor red light stimulated the transcription of the genes for superoxide dismutase (*sod, lmo1439*), catalase (*kat; lmo2785*) or of *lmo0799* itself and although SigB is involved in oxidative stress resistance of *L. monocytogenes* [89], such stress is not a typical inducer of the SigB system. Therefore the mechanism of *L. monocytogenes* SigB acticvation by red light remains unknown.

**Figure 7:**
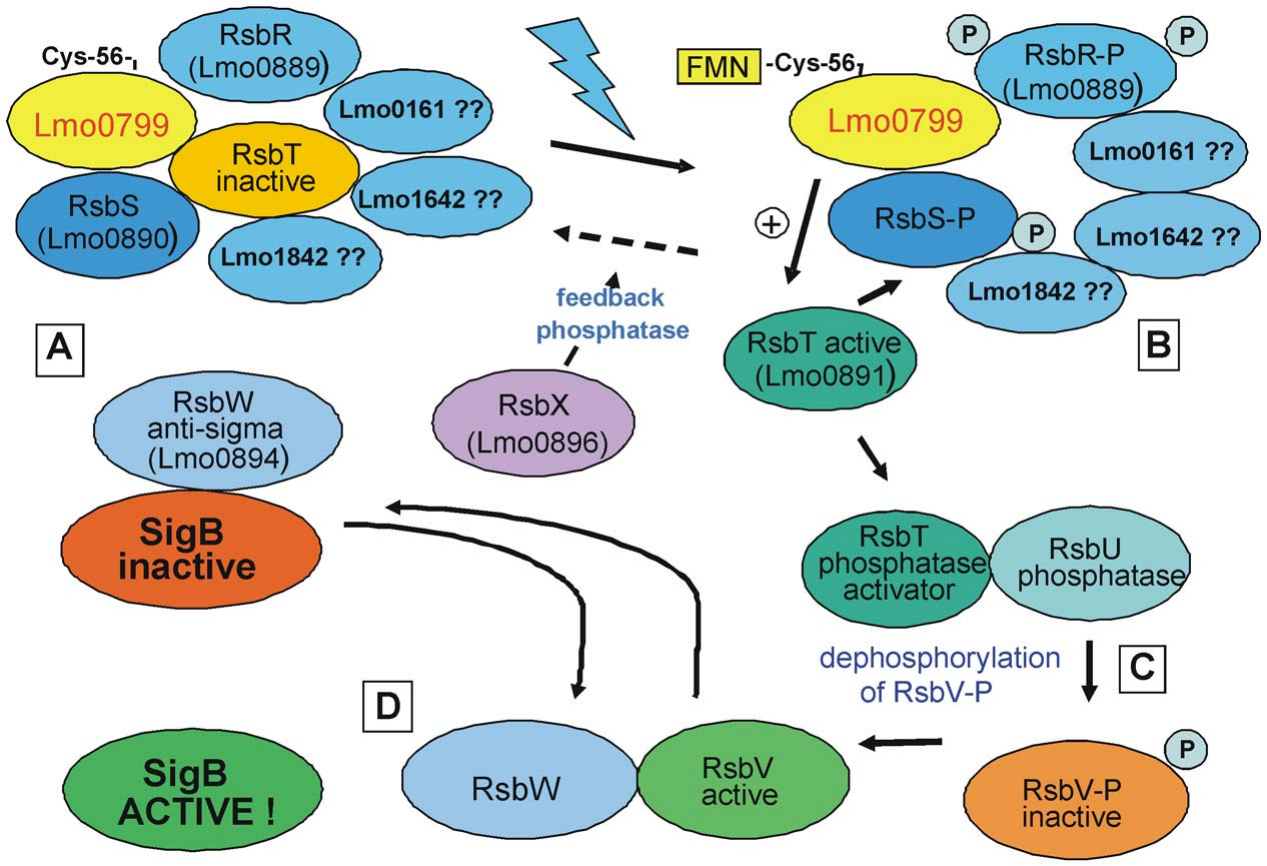
Hypothetical and simplified model of SigB activation by blue light. (A) Hypothetical complex of Lmo0799, RsbR and its putative paralogues (Lmo0161, Lmo1642, Lmo1842), RsbS and RsbT in the dark state. SigB is sequestered by the anti-sigma factor RsbW, which also keeps RsbV inactive by phosphorylation. (B) complex after blue-light driven FMN-Lmo0799 photoadduct formation, activating the kinase function of RsbT, which phophorylates RsbS and RsbR and is released from the complex, binds and activates RsbU. (C) dephosphorylation of RsbV-P by activated RsbU, (D) dephosphorylated RsbV binds RsbW and active SigB is released. The feedback phosphatase RsbX is believed to limit the activation of the system. Further explanations in the text. The graph does not intend to reflect the stoichiometry of the individual proteins, which presumably are present in multiple copies, nor the true architecture of the hypothetical complex. Modified from the scheme proposed for *B. subtilis* YtvA [47].

As has been mentioned in the introduction, in *B. subtilis* different environmental stresses, ultimately evoking the activation of SigB, are integrated by a dynamic supramolecular complex, the stressosome [38], [39], [42], [43], [44], [45]. For *B. subtilis* it has also firmly been established that YtvA is part of its stressosome [47], this raised the question if *L. monoyctogenes* could have a stressosome too. Homologues of the *B. subtilis* stressosome constituents RsbS, RsbT, RsbRA and for the downstream components RsbU, RsbV and RsbW as well as for the feedback phosphatase RsbX have been identified in *L. monocytogenes* and it has been shown that their genomic and transcriptional organization is identical to that of *B. subtilis* [27], [82]. We identified Lmo0799 as a YtvA homologue and hence as a RsbR paralogue in *L. monocytogenes*. A BLAST search in the complete genome of *L. monocytogenes* EGD-e with the protein sequences of *B. subtilis* RsbRA and the RsbRA paralogues RsbRB (YkoB), RsbRC (YojH) and RsbRD (YqhA) as query confirmed Lmo0889 as the RsbRA homologue. Some homology (about 25 percent identity/46 percent similarity) to RsbRB-D was only found for Lmo0161. When RsbR (Lmo0889, 278 amino acids) of *L. monocytogenes* itself was used as a query sequence, again homology to Lmo0161 was found, but now also to Lmo1642 (267 amino acids, 21/52 percent) and Lmo1842 (274 amino acids, 23/45 percent). These homologies extended over the whole length of all three proteins, which also in size were very similar to RsbR (Lmo0889). Domain analysis with SMART [90] and InterPro [91] revealed that all three proteins had a C-terminal STAS domain, typical for the RsbR protein family, but a canonical Rsbr-N domain could not be identified. In a comparative analysis of *B. subtilis* RsbRB-D also only a C-terminal STAS domain could be identified by SMART and InterPro (results not shown). Figure S2 shows the domain analyses for *L. monocytogenes*, together with a sequence alignment of *B. subtilis* RsbRA-D. Future experiments have to show if these proteins are functional RsbR paralogues. Figure 7 shows a hypothetical and simplified, with respect to the RsbR paralogues purely speculative model of the SigB activation via blue light and Lmo0799.

Although the mechanism by which red light induces the SigB regulon in *L. monocytogenes* is unknown, there is obviously some crosstalk or interference with the Lmo0799-dependent blue-light triggered mechanism. As shown in figures 2BD and 3BD red light induced the transcription of SigB-dependent genes and of *sigB* in the wild type only when additional salt stress was imposed whereas in the absence of Lmo0799, i.e. in the *Δ0799* mutant, red light alone lead to a significant up regulation. This shows that Lmo0799 or the hypothetical Lmo0799-containing complex inhibited the transduction of the red-light signal by an unknown mechanism. It will be interesting to see if such an effect also occurs in *B. subtilis* where the red-light signal obviously is transduced in a different way.

Figure 4 shows that the transcription profile of the virulence regulator *prfA* was very similar to that obtained for SigB-dependent genes, whereas light or dark had no effect on the expression of the PrfA-dependent gene *plcA. PrfA* can be transcribed as a bicistron together with the upstream *plcA* gene and monocistronically from two promoters directly upstream of *prfA* [14], [15], one of which (P2) is under SigB control [15], [16], [32]. Since no increase in *plcA* transcription was seen here, salt and light caused an increase solely in monocistronic *prfA* mRNA due to an activation of SigB, this is in line with previous reports on the impact of SigB on *prfA* [15], [16], [31], [32], [34]. However, elevated levels of *prfA* mRNA do not always lead to a corresponding increase in the transcription of PrfA-dependent virulence genes, e.g. *plcA*, rather this is also determined by the amount and activity of the PrfA protein which is subject to a very complicated and not completely understood regulation. It has been shown that two S-adenosylmethionine riboswitches control the translation of *prfA* mRNA [92]. Furthermore, there is evidence that an unknown cofactor regulates PrfA activity [15], [16], alternatively or in addition, the transport of sugars via phosphotransferase systems has a negative effect on PrfA activity, reviewed in [17]. So it seems that under the conditions used in our experiments the increase in *prfA* transcription does not translate into more and active PrfA protein.

The results were different for the internalins A and B. It has previously been reported that transcription of *inlA* and *inlB* was not only positively regulated by PrfA, but that it was also diminished in mutants lacking SigB [32], [33], [34], [79], [81] and increased when SigB was activated [93]. Furthermore, it has been shown that invasion of Caco-2 enterocytes is largely dependent on SigB [94]. Our results show (Figure 5A) that the transcription profiles of *inlA* and *inlB* in the dark or after exposure to blue light were identical to those observed by us for SigB-dependent genes. Invasion experiments with Caco-2 enterocytes showed that two hours after the addition of *L. monocytogenes* the number of intracellular bacteria was twice as high after pretreatment of the wild type with blue light and 0.3 M NaCl as without light or for the Δ*0799* blue-light receptor mutant (Figure 5B). Potential differences in intracellular replication during this short time are not supposed to have a significant effect on the results. Also for the internalins A and B post-transcriptional control of the protein levels has been reported [95], [96], this could be a reason why the slight transcriptional induction in the Δ*0799* mutant did not result in a corresponding invasiveness, however, experimental data supporting this assumption are lacking. If this light-induced invasiveness has a role in the natural infection process, preparing the bacteria for a potential ingestion by a host organism, will be difficult to test. It could be an accessory factor, contributing to the postulated major effect of the intestinal environment on invasiveness [22].

Light is one of the environmental factors which may influence flagella-mediated movement of bacteria [63]. We observed (Figure 6) a drastic reduction of swimming motility when *L. monocytogenes* wild type was exposed to blue light at 27°C, this inhibition was completely relieved in the *Δ0799* blue-light receptor and *ΔsigB* mutants. Most *L*. *monocytogenes* strains, including EGD-e, are non-motile at temperatures around 37°C because at this temperature the flagella- and motility gene cluster [72], [97] is repressed by a system comprising several factors [97], [98]. There is no evidence that blue-light activated Lmo0799 can directly interfere with this regulatory system, however, it has previously been described that the flagella- and motility cluster was transcriptionally down regulated at 24°C in salt-stressed *L. monocytogenes* and that a Δ*sigB* strain showed increased swarming. From their data the authors concluded that SigB negatively regulates motility in an indirect way [80], a negative regulation of flagella- and motility-associated genes by SigB has also been reported by others [34], [79]. Here we could show that blue light activated SigB in a Lmo0799-dependent manner and that inhibition of motility by blue light was SigB-dependent. Therefore the observed inhibition of swimming motility by blue light in the wild type most presumably was also due to transcriptional silencing of the flagella- and motility cluster via Lmo0799 and SigB. An experiment-based explanation for the fact that red light had no inhibitory effect here, although it also induced SigB-dependent genes, is lacking. It could be that this effect was transient and faded away during the 18 h duration of the swimming motility assay, whereas the Lmo0799-mediated activation by blue light was persistent. A persistent SigB activation by blue light has been mentioned for YtvA of *B. subtilis* (59). Alternatively, one could assume that the inhibition of motility requires both SigB and blue light-activated Lmo0799, the latter one missing under red light illumination.

Intense blue and, to a lesser extent, red light can result in oxidative damage to cells by the light-driven formation of reactive oxygen intermediates [86], [87], [88]. Therefore the benefit of evolving light sensing systems also in non-phototrophic bacteria seems obvious, possibly enabling them to activate appropriate defense systems at an early time point, i.e. already at low light intensity before massive damage will occur [67]. However, in our experiments we did not obtain evidence for the up regulation of oxidative stress defense genes in *L. monocytogenes* by moderate illumination. Furthermore, light would be a suitable signal for upcoming osmotic stress, caused by water evaporation under sun light. The illuminance we used in our experiments, i.e. 30 microeinsteins/m^2^/s of blue or red light, cannot exactly be compared to day- or sunlight because natural light contains the whole visible spectrum with varying proportions of the different wavelengths and a maximum in the blue-green range [99]. The experimental conditions used here correspond to approximately the illuminance in the half-shade at noon on an overcast day or to about 1/30 of full sunlight at mid-latitude [99]. Our results show that visible light had an impact not only on osmotic stress defense factors of *L. monocytogenes* but on other functions too, e.g. invasiveness and swimming motility. Furthermore, it has previously been reported that the large SigB regulon of *L. monocytogenes* also comprises genes for transport, metabolism and protein synthesis [32], [79], [80], [81]. Therefore one can assume that in natural environments, depending on their exposure to light, all these processes may undergo cyclic fluctuations. These will not be genuinely circadian because prokaryotic endogenous clocks have so far been found in cyanobacteria only [100], rather their periodicity will be determined by day length and hence by season, weather and geographical latitude. Furthermore, it has been shown that the specific transcriptional regulation by SigB varies among *L. monocytogenes* strains [81], therefore also the dimension of light effects will vary accordingly.

## Materials and Methods

### General techniques

PCR amplifications, cloning procedures, isolation of chromosomal DNA, and DNA manipulations were carried out according to standard procedures [101]. DNA sequencing was carried out by Seqlab GmbH (Germany).

### Bacterial strains, plasmid, and cell line

*L. monocytogenes* Sv1/2a EGD-e (ATCC BAA-679) was obtained from T. Chakraborty (University of Giessen, Germany) whom we also thank for the isogenic *sigB*-deletion mutant (Δ*sigB*) [79]. *E. coli* strain TG1 and plasmid pG^+^host4 [102] were kindly provided by E. Maguin (INRA Jouy en Josas, France). Human colon epithelial cells (Caco-2 cells) were obtained from the American Type Culture Collection (ATCC HTB-37) and were cultured at 37°C and 5% CO_2_ in RPMI 1640 (Gibco, Germany) supplemented with 10% heat-inactivated fetal calf serum (FCS) (Biochrom KG, Germany).

### Media and growth conditions

*L. monocytogenes* was grown in brain heart infusion (BHI, Difco, Germany) at 37°C. Cultivation of *E. coli* was carried out in Luria-Bertani (LB, 10 g/l peptone, 5 g/l yeast extract, 10 g/l NaCl) medium at 37°C. For transformation experiments media were supplemented with erythromycin to final concentrations of 300 µg/ml for *E. coli* or 10 µg/ml for *L. monocytogenes*. For growth tests of *L. monocytogenes* 300 µl of an overnight culture in BHI were diluted into 10 ml prewarmed BHI and shaken at 190 rpm and 37°C. The optical density of the cultures was recorded every hour with a photometer (Ultrospec, Amersham Biosciences, Germany) at 600 nm in 1 cm cuvettes.

### Mutant construction

For construction of the in frame deletion mutant (Δ*lmo0799*) PCR-amplified fragments of ∼300 bp from the 5′ and 3′ region of *lmo0799* were cloned into the temperature-sensitive integration vector pG^+^host4 and transformed into *E. coli* TG1 [102]. *L. monocytogenes* EGD-e was transformed with the plasmid construct and plasmid integrants were selected at the non-permissive temperature of 42°C on erythromycin-containing BHI agar. To obtain the deletion, the mutant strains were subcultured twice for 24 h in BHI without antibiotic at 30°C. At this temperature the plasmid origin of replication is fully active which favors plasmid excision [102]. Serial dilutions of the subcultures were plated on BHI without antibiotic and erythromycin-sensitive clones were identified by replica-plating on erythromycin-containing medium. Plasmid loss and deletion was confirmed by PCR and DNA sequencing (data not shown). Oligonucleotides used for mutant construction are listed in table S1.

### RNA isolation and preparation of samples for infection

For transcription analysis and infection experiments *L. monocytogenes* was precultured in BHI at 37°C under aerobic conditions without light, i.e. all culture vessels were wrapped in two layers of aluminium foil. For dark controls, all manipulations were carried out under low-intensity, diffuse infrared illumination (Osram Opto SFH4730 LED, 3 W, λ = 850 nm), using Dipol D2MVSL night vision goggles (Gross, Germany) for observation. Overnight precultures were diluted into fresh medium and grown in the dark at 37°C to an OD_600_ ∼0.9. For salt stress experiments 3.0 M sterile NaCl was added to the respective cultures to a final concentration of 0.3 M. Cultures were split into two 250 ml cell culture bottles, one wrapped twice in aluminium foil, the other exposed to blue (light-emitting diode, peak wavelength λ = 455 nm, Luxeon Star LXHL-MRRD, 1 W) or red (peak wavelength λ = 625 nm, Luxeon LXHL-MD1D, 1 W) light with an illuminance of 30 microeinstein/m^2^/s for 10 min, both samples were kept at 37°C. Samples for infection were washed twice with 1xPBS in the dark and stored in 1xPBS/20% glycerol (v/v) as 1 ml aliquots at −80°C in a light-tight container. Samples for RNA isolation (10 ml) were centrifuged in the dark for 10 min at 4°C and cell pellets were frozen in liquid nitrogen. RNA was prepared using the Seqlab RNA Mini Kit (Seqlab, Germany). Residual DNA was removed by Turbo DNAse treatment (Applied Biosystems/Ambion, USA). All RNA isolations were repeated at least three times.

### Real-time qRT-PCR

Real-time quantitative reverse transcriptase PCR (qRT-PCR) was performed on total RNA isolated. The absence of DNA from RNA samples was verified by PCR prior to reverse transcription, using *rpoB* -specific primers. 5 µg of total RNA was reverse transcribed with random hexamers and SuperScript II™ Reverse Transcriptase (Invitrogen, USA) according to the manufacturer's instruction. qRT – PCRs were performed in a total volume of 25 µl using PerfeCTa™ SYBR® Green FastMix™ ROX (Quanta Biosciences, USA) and an Applied Biosystems StepOne™ Plus cycler. The housekeeping gene *rpo*B served as an internal standard [103], [104]. All transcription analyses were done on at least three independent biological samples and with four technical replicates each. The primers used are listed in Table S1.

### Motility assays

Swimming motility of the wild type, of the *Δlmo0799* and of the Δ*sigB* mutant strain was tested on semi-solid BHI agar (0.3%) plates, inoculated with 2 µl of mid-log bacterial cultures (OD_600_ ∼0.7) grown in BHI at 37°C. Plates were sealed and incubated for 18 h at the indicated temperature in the complete dark or under exposure to blue (λ = 455 nm) or red (λ = 625 nm) light, respectively, at an illuminance of 30 microeinstein/m^2^/s. Motility was quantified as the diameter of the swimming colony.

### Infection assays with Caco-2 enterocytes

Infection assays were performed in triplicate using 24-well tissue culture plates (Greiner Bio, Germany), seeded with 3×10^5^ Caco-2 cells per well and then incubated at 37°C and with 5% CO_2_. After 24 hours the wells were checked for a semi-confluent monolayer, the cells were washed with 1xPBS containing Ca/Mg and infected using low-intensity, diffuse infrared illumination (LED, λ = 850 nm) and Dipol D2MVSL night vision goggles (Gross, Germany) for observation. RPMI 1640 as medium and an m.o.i. of 20 for *L. monocytogenes* EGD-e wild type or its *Δlmo0799* mutant were used, the bacteria had been preincubated at 37°C with 0.3 M NaCl either in the dark or under exposure to blue light (λ = 455 nm) for 10 min at an illuminance of 30 microeinstein/m^2^/s. The plates were wrapped in two layers of aluminium foil and incubated for 1 h at 37°C in a 5% CO_2_ atmosphere. After 1 h the infection medium was replaced by RPMI with 100 µg ml^−1^ gentamicin and incubation was continued for another hour. The experimental steps after the addition of gentamicin were carried out at ambient light. At the end of the infection period cells were washed twice with 1× PBS and lysed with distilled water for 30 min on ice. Serial dilutions of the bacteria-containing lysate were plated in triplicate on BHI plates which were incubated for 24 h at 37°C. Colonies were counted and absolute (CFU/ml) and relative (percent) infection rates were calculated. All infection experiments were repeated at least three times.

### Sequence data acquisition, bioinformatics and data analysis

Protein sequences were obtained from the sequenced genomes of *L. monocytogenes* EGD-e [72] and *B. subtilis* 168 [76], respectively, using the Genolist website at the Pasteur Institute Paris (http://genolist.pasteur.fr/ListiList; http://genolist.pasteur.fr/SubtiList). Homology searches, using BLAST [105], were performed on these websites, for *L. ivanovii* at http://genolist.pasteur.fr/LivaList and for other listerial and bacterial genomes at NCBI (http://www.ncbi.nlm.nih.gov/sutils/genom_table.cgi). Sequence alignments were done with ClustalW2 [106] at the European Bioinformatics Institute (EBI, UK) facility (http://www.ebi.ac.uk/Tools/clustalw2/index.html), using the default settings. For 3-D modelling of proteins the SWISS-MODEL server (http://swissmodel.expasy.org/workspace/) at the Swiss Institute of Bioinformatics (SIB) was used in the automated mode [107], [108]. Protein domain analysis was performed with SMART6 [90] at http://smart.embl-heidelberg.de and also using InterPro [91] at http://www.ebi.ac.uk/interpro/. For the analysis of the results from qRT-PCR experiments the Applied Biosystems' StepOne™ Software was used.

## Supporting Information

Table S1 Oligonucleotide primers used in this study.
(PDF)

Figure S1 Effect of blue and red light on *trxA*, *lmo0799*, *kat* and *sod* expression. (A, B) Transcription analysis by qRT-PCR of *trxA* for wild type, Δ*0799* and *ΔsigB* mutants. (C, D) Transcription analysis by qRT-PCR of *lmo0799, kat* and *sod* for wild type. The strains were grown at 37°C in BHI. Cells were harvested in mid-log phase (OD_600_ ∼0.9) and exposed for 10 min to blue (455 nm) or red (625 nm) light as described in material and methods. The results from the qRT-PCR analysis, obtained with a StepOnePlus Real-Time PCR system (Applied Biosystems Inc.) were normalized using *rpoB* as an internal standard [103], [104] and expressed as fold change with the values for wild type without light set as 1.0. Calculations were performed with the StepOne Software v2.1 (Applied Biosystems Inc.). Means and standard deviations from three independent biological samples and four technical replicates per sample.
(PDF)

Figure S2 Domain analysis and alignment of RsbR paralogues. (A) Graphical representation of domains identified by SMART (http://smart.embl-heidelberg.de) [92] in RsbR (Lmo0889), Lmo0161, Lmo1642, Lmo1842, Lmo0799 and RsbS (Lmo0890) of *L. monocytogenes* EGD-e. The protein sequences were obtained from the ListiList website (http://genolist.pasteur.fr/ListiList). The LOV domain is not implemented in SMART, therefore this domain of Lmo0799 is depicted as PAS-PAC (Per-Arnt-Sim signal sensor domain/PAS-associated domain). LOV domains are a subfamily of the PAS superfamily [51]. (B) Amino acid alignment of RsbRA-D of *B. subtilis*, RsbR and putative paralogues of *L. monocytogenes* EGD-e, using ClustalW2 [106]. The prefix Bs denotes proteins from *B. subtilis*, Lm from *L. monocytogenes*. Asterisks below the sequence indicate identical, double points very similar amino acids. The C-terminal STAS domain is indicated, the crucial threonines (T171/T205 in *B.s.* RsbRA, T175/T209 in *L.m.* RsbR) are highlighted in yellow, negatively charged amino acids (aspartate D, glutamate E) in the putative RsbR paralogues in blue. Further explanation in the text. The *B. subtilis* protein sequences were from SubtiList (http://genolist.pasteur.fr/SubtiList), the *L. monoytogenes* sequences from ListiList (http://genolist.pasteur.fr/ListiList). The GenBank accession nos. for the respective genome sequences are AL591824 (*L.m*.) and AL009126 (*B.s*.).
(PDF)
