# Supplementary material for: Blue and Red Light Modulates SigB-Dependent Gene Transcription, Swimming Motility and Invasiveness in Listeria monocytogenes
Source: PLoS One. 2011 Jan 11;6(1):e16151. doi: 10.1371/journal.pone.0016151 (PMC3019169; doi:10.1371/journal.pone.0016151)
Supplement: Table S1 — Oligonucleotide primers used in this study. (PDF) [file pone.0016151.s001.pdf]

Table S1. Oligonucleotide primers used in this study

Oligoname	Sequence (5' -> 3')
Mutant construction	Introduced restriction sites underlined
0799 chk DO	GTC GTC GGC GTC TTT GTT TCG
0799 chk UP	CGG ACA GCT AAT CAC TTC ATC TTT ATC
0799 DO F - HindIII	CAG CAC AAT CAA AGA AGC TTT AC
0799 DO R - Pst1	CAG CCA ATC TGC AGG AAA TTG
0799 UP F - BamH1	CCA ATG TTT GAT TGG ATC CCG TCA TC
0799 UP R - HindIII	CGA ATT GTG GAT AAG CTT TCA TCT GC
pG+host4	flanking multiple cloning site
M14	TTG TAA AAC GAC GGC CAG TGA
RedR	ACA GGA AAC AGC TAT GAC CAT
qRT - PCR primers	(products are ca. 150 bp)
arsC - F	TAT TCG AAG TGC CAT TGC TG
arsC - R	ACT TTC AGG TGG CTC GAT TG
bsh - F	TTG CTG CTG TGA TGG AAA AC
bsh - R	ATT CAA AGG GGG TCA CAT TG
ctc - F	TGG CGG TGT ACT TCA ACA AA
ctc - R	TCT TTG TTT TCC GGA AGG TC
inlA-qRT-F	TGT GAC TGG CGC TTT AAT TG
inlA-qRT-R	TCC AAT AGT GAC AGG TTG GCT A
inlB-qRT-F	AAA AGC AAG ATT TCA TGG GAG A
inlB-qRT-R	TCG GAG GTT TAG GTG CAG TT
0799 qRT - F	TAC ATG GAT CAC GGC AAA GA
0799 qRT - R	GCG AAA TTC CGG TGA TGA TA
Lmo1233-F	TTG AAC AAG AAA CTA GCG AAG G
Lmo1233-R	TTC ATC TAC GTC CAT TTT GAC G
lmo1433_3	AAC TAA TGC CGC TAC GAA ATC

lmo1433_5	CTC CCA GAT TCT GTC GTT TTT
lmo1439_3	TTC GAT ATA TTC AGG ACG ACG
lmo1439_5	TCT ACA GCT AAC CAA GAT TCT C
lmo2478_3	TAC TCA GCT CCA AAT TGT TTC G
lmo2478_5	TTA ATG ATT GAA CGC GGT GTA C
lmo2770_3	TTC GCG AAG GTA GAT GAT TTC
lmo2770_5	AAA AAC ACA GAT CCT TTG CGC
lmo2785_3	AGC GTC ATT GTT CCT ACG T
lmo2785_5	TGA AAA TGG AGA CTA TCC TG
opuCD-qRT-F	CTT ACG GAG TTA TCT TCG CAG C
opuCD-qRT-R	ATT GCT AGT GCC GGA ATT GT
plcA – F	GCA GCA AAA CAG CAA CGA TA
plcA - R	CCG CGG ACA TCT TTT AAT GT
prfA – F	ACGGGAAGCTTGGCTCTATT
prfA - R	TGCGATGCCACTTGAATATC
rpoB-qRT-F	CAC CCT GAA GCT CCA TTT GT
rpoB-qRT-R	ACA CGA CGA ACC CAG ATT TC
SigB-qRT-F	CGC CGA ATC AAA GAG TTA GG
SigB-qRT-R	CTT TTT CCC ATT TCC ATT GCT TC
