# Supplementary material for: Blue and Red Light Modulates SigB-Dependent Gene Transcription, Swimming Motility and Invasiveness in Listeria monocytogenes
Source: PLoS One. 2011 Jan 11;6(1):e16151. doi: 10.1371/journal.pone.0016151 (PMC3019169; doi:10.1371/journal.pone.0016151)
Supplement: Figure S1 — Effect of blue and red light on trxA, lmo0799, kat and sod expression. (A, B) Transcription analysis by qRT-PCR of trxA for wild type, Δ0799 and ΔsigB mutants. (C, D) Transcription analysis by qRT-PCR of lmo0799, kat and sod for wild type. The strains were grown at 37°C in BHI. Cells were harvested in mid-log phase (OD600 ∼0.9) and exposed for 10 min to blue (455 nm) or red (625 nm) light as described in material and methods. The results from the qRT-PCR analysis, obtained with a StepOnePlus Real-Time PCR system (Applied Biosystems Inc.) were normalized using rpoB as an internal standard [103], [104] and expressed as fold change with the values for wild type without light set as 1.0. Calculations were performed with the StepOne Software v2.1 (Applied Biosystems Inc.). Means and standard deviations from three independent biological samples and four technical replicates per sample. (PDF) [file pone.0016151.s002.pdf]

Blue and red light modulates SigB-dependent gene transcription, swimming motility and invasiveness in *Listeria monocytogenes*

Nicolai Ondrusch, Jürgen Kreft*

Supporting Information Figure S1

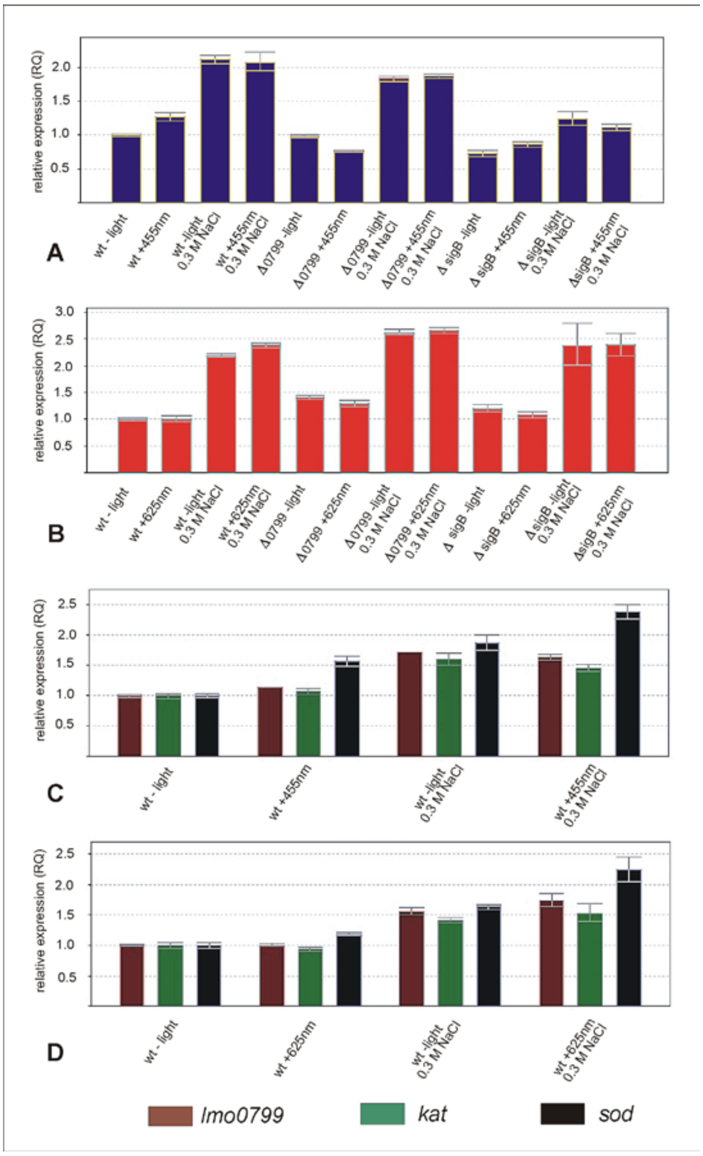

Figure S1. Effect of blue and red light on *trxA*, *lmo0799*, *kat* and *sod* expression. (A, B) Transcription analysis by qRT-PCR of *trxA* for wild type, Δ*0799* and *ΔsigB* mutants. (C, D) Transcription analysis by qRT-PCR of *lmo0799*, *kat* and *sod* for wild type. The strains were grown at 37 °C in BHI. Cells were harvested in mid-log phase (OD_{600} ~ 0.9) and exposed for 10 min to blue (455 nm) or red (625 nm) light as described in material and methods. The results from the qRT-PCR analysis, obtained with a StepOnePlus Real-Time PCR system (Applied Biosystems Inc.) were normalized using *rpoB* as an internal standard [103,104] and expressed as fold change with the values for wild type without light set as 1.0. Calculations were performed with the StepOne Software v2.1 (Applied Biosystems Inc.). Means and standard deviations from three independent biological samples and four technical replicates per sample.
