# Supplementary material for: Blue and Red Light Modulates SigB-Dependent Gene Transcription, Swimming Motility and Invasiveness in Listeria monocytogenes
Source: PLoS One. 2011 Jan 11;6(1):e16151. doi: 10.1371/journal.pone.0016151 (PMC3019169; doi:10.1371/journal.pone.0016151)
Supplement: Figure S2 — Domain analysis and alignment of RsbR paralogues. (A) Graphical representation of domains identified by SMART (http://smart.embl-heidelberg.de) [92] in RsbR (Lmo0889), Lmo0161, Lmo1642, Lmo1842, Lmo0799 and RsbS (Lmo0890) of L. monocytogenes EGD-e. The protein sequences were obtained from the ListiList website (http://genolist.pasteur.fr/ListiList). The LOV domain is not implemented in SMART, therefore this domain of Lmo0799 is depicted as PAS-PAC (Per-Arnt-Sim signal sensor domain/PAS-associated domain). LOV domains are a subfamily of the PAS superfamily [51]. (B) Amino acid alignment of RsbRA-D of B. subtilis, RsbR and putative paralogues of L. monocytogenes EGD-e, using ClustalW2 [106]. The prefix Bs denotes proteins from B. subtilis, Lm from L. monocytogenes. Asterisks below the sequence indicate identical, double points very similar amino acids. The C-terminal STAS domain is indicated, the crucial threonines (T171/T205 in B.s. RsbRA, T175/T209 in L.m. RsbR) are highlighted in yellow, negatively charged amino acids (aspartate D, glutamate E) in the putative RsbR paralogues in blue. Further explanation in the text. The B. subtilis protein sequences were from SubtiList (http://genolist.pasteur.fr/SubtiList), the L. monoytogenes sequences from ListiList (http://genolist.pasteur.fr/ListiList). The GenBank accession nos. for the respective genome sequences are AL591824 (L.m.) and AL009126 (B.s.). (PDF) [file pone.0016151.s003.pdf]

Blue and red light modulates SigB-dependent gene transcription, swimming motility and invasiveness in *Listeria monocytogenes*

Nicolai Ondrusch, Jürgen Kreft*

Supporting Information Figure S2

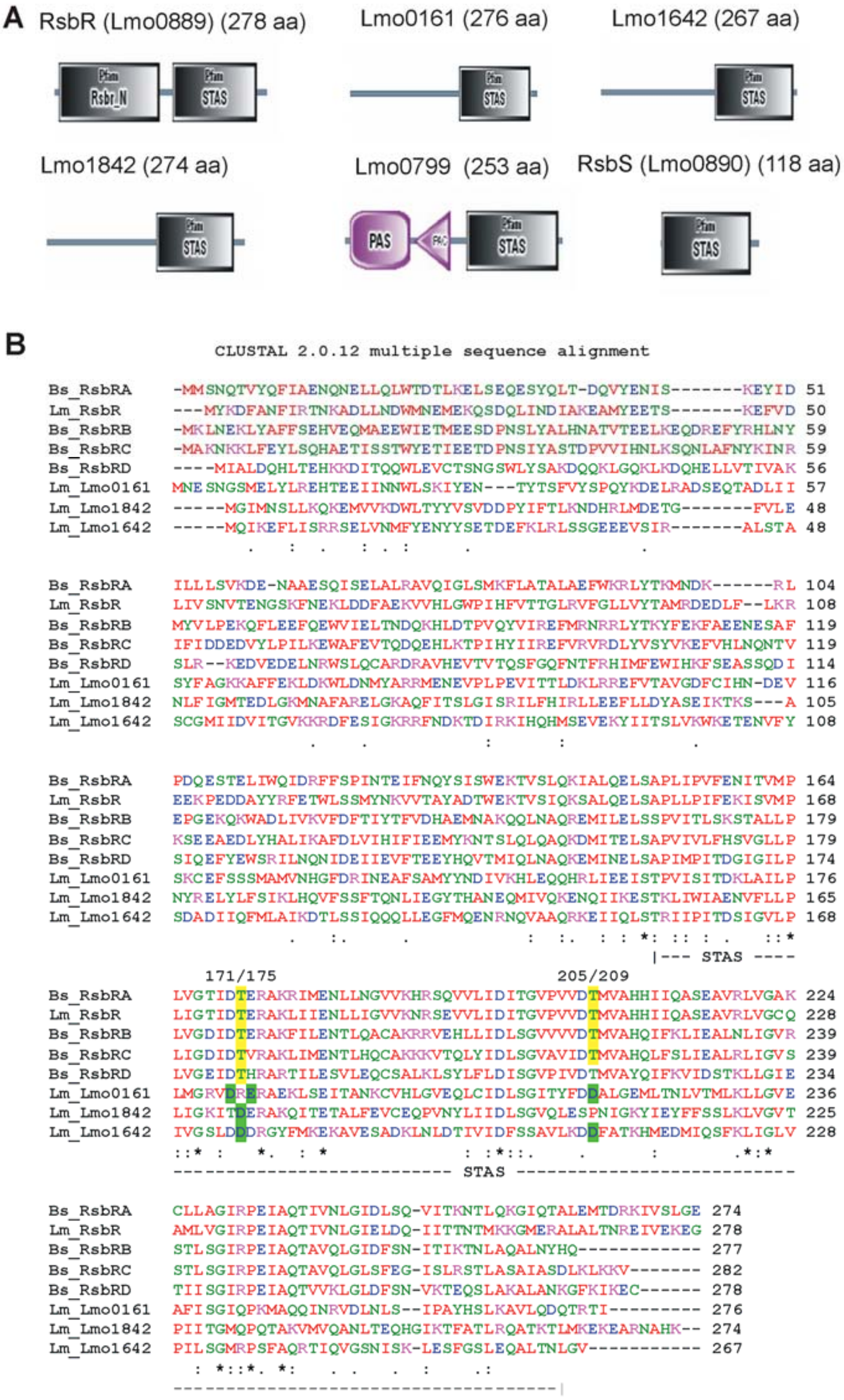

Figure S2. Domain analysis and alignment of RsbR paralogues. (A) Graphical representation of domains identified by SMART (http://smart.embl-heidelberg.de) [92] in RsbR (Lmo0889), Lmo0161, Lmo1642, Lmo1842, Lmo0799 and RsbS (Lmo0890) of *L. monocytogenes* EGD-e. The protein sequences were obtained from the ListiList website

(http://genolist.pasteur.fr/ListiList). The LOV domain is not implemented in SMART, therefore this domain of Lmo0799 is depicted as PAS-PAC (Per-Arnt-Sim signal sensor domain / PAS-associated domain). LOV domains are a subfamily of the PAS superfamily [51]. (B) Amino acid alignment of RsbRA-D of *B. subtilis*, RsbR and putative paralogues of *L. monocytogenes* EGD-e, using ClustalW2 [110]. The prefix Bs denotes proteins from *B. subtilis*, Lm from *L. monocytogenes*. Asterisks below the sequence indicate identical, double points very similar amino acids. The C-terminal STAS domain is indicated, the crucial threonines (T171/T205 in *B.s.* RsbRA, T175/T209 in *L.m.* RsbR) are highlighted in yellow, negatively charged amino acids (aspartate D, glutamate E) in the putative RsbR paralogues in blue. Further explanation in the text. The *B. subtilis* protein sequences were from SubtiList (http://genolist.pasteur.fr/SubtiList), the *L. monoytogenes* sequences from ListiList (http://genolist.pasteur.fr/ListiList). The GenBank accession nos. for the respective genome sequences are AL591824 (*L.m.*) and AL009126 (*B.s.*).
